# Antioxidant Activity of 3-[*N*-(Acylhydrazono)ethyl]-4-hydroxy-coumarins

**DOI:** 10.3390/molecules21020138

**Published:** 2016-01-23

**Authors:** Antigoni Kotali, Despina A. Nasiopoulou, Constantinos A. Tsoleridis, Philip A. Harris, Christos A. Kontogiorgis, Dimitra J. Hadjipavlou-Litina

**Affiliations:** 1 Laboratory of Organic Chemistry, Department of Chemical Engineering, Aristotle University of Thessaloniki, Thessaloniki 54124, Greece; dnasiopoulou@yahoo.gr; 2 Laboratory of Organic Chemistry, Department of Chemistry, Aristotle University of Thessaloniki, Thessaloniki 54124, Greece; tsolerid@chem.auth.gr; 3 GlaxoSmithKline, 1250 South Collegeville Road, P. O. Box 5089, Collegeville, PA 19426-0989, USA; philip.a.harris@gsk.com; 4 Department of Pharmaceutical Chemistry, School of Pharmacy, Aristotle University of Thessaloniki, Thessaloniki 54124, Greece; ckontogi@med.duth.gr (C.A.K.); hadjipav@pharm.auth.gr (D.J.H.-L.)

**Keywords:** antioxidants, 3-acetyl-4-hydroxycoumarin, acyl hydrazones, DPPH, trolox, lipid peroxidation, soybean lipoxygenase

## Abstract

A series of 3-acylhydrazono-4-hydroxycoumarins were synthesized via condensation of 3-acetyl-4-hydroxycoumarin with appropriate hydrazides. The structures of the newly-synthesized compounds were characterized by spectral and elememental analysis or HRMS measurements. Their antioxidant properties were evaluated by using scavenging effects on 2,2-diphenyl-1-picrylhydrazyl (DPPH) radical as well as inhibition of lipid peroxidation. Moreover, their ability to inhibit *in vitro* soybean lipoxygenase has been investigated. They were found to be capable of rapid inactivation of alkylperoxy radicals.

## 1. Introduction

3-Substituted-4-hydroxycoumarins constitute an important class of heterocycles, which occur widely among natural products and have interesting biological properties and importance in medicine. They have been reported to exhibit a variety of pharmacological activity as antibacterial, [1,2] antitumor, [3] activity against HIV virus, [4] antithrombotic, [5,6,7,8], as well as antioxidant activity [9,10]. Coumarins are one of the most important secondary metabolites of plants and are known as naturally occurring benzo-α-pyrone derivatives from metabolism of phenylalanine [11]. More than 1000 different types of coumarins have been isolated from natural sources. Robustic acid, [12] ferulenol and its analogues, [13,14] as well as the two sesquiterpenecoumarins isolated from *Ferula pallida* [15], are characteristic examples of 4-hydroxycoumarins, which have been isolated as natural products. Furthermore, the 4-hydroxycoumarin moiety has been the molecular template for the synthesis of a variety of analogues with important biological activity. Warfarin is a synthetic coumarin, which is widely used as anticoangulant, [16] whereas aminocoumarin analogues, such as novobiocin, chlorobiocin, coumermycin, and simocyclinone are potent antibiotics [17,18,19]. Furthermore, the importance of hydrazone derivatives is well known because of their use as synthons in organic synthesis [20,21] as well as because of their biological properties. They have been reported to possess among others anticonvulsant, antidepressant, analgestic, anti-inflammatory, antimicrobial, antimalarial, antitumoral, antileukemic, antiviral, antitubercular, as well as antioxidant activity [22,23,24,25,26].

It is well known that the design of biological substrates with antioxidant activity to be used for disease treatment or as food additives, as well as oxidative stress, have attracted many researchers’ interest. The potential activity of both coumarin, as well as hydrazone derivatives, as antioxidant agents prompted us to synthesize a series of new coumarin analogues bearing the 3-acylhydrazono functionality and a 4-hydroxy group on the coumarin ring. The combination of the pharmacophores of two different biologically-active compounds in the same molecule could lead to a new product exhibiting combined activity.

The formation of Reactive Oxygen Species (ROS) is characteristic of aerobic organisms that can normally defend themselves against these highly reactive species. However, in many pathophysiological conditions the excessive production of ROS overwhelms the natural antioxidant defense mechanisms. This imbalance is termed oxidative stress, which has been associated with the inflammation process. ROS, like superoxide radical anion, hydrogen peroxide and hydroxyl radical, are produced during the inflammation process by phagocytic leukocytes. Moreover, these reactive species are involved in the biosynthesis of prostaglandins and in the cycloxygenase- and lipoxygenase-mediated conversion of arachidonic acid. The rates of ROS production are increased in most pathophysiological conditions [27]; therefore, it is evident that the treatment of various diseases could benefit from the use of drugs that combine antioxidant and anti-inflammatory activity.

Thus, based on the above literature findings and on our interest in coumarin [28,29,30,31,32] and hydrazone derivatives [20,21], as well as in the biological activity of small molecules [23,29], we present here the synthesis and structural characterization of a series of 3-acylhydrazono-substituted 4-hydroxycoumarins, as well as their *in vitro* antioxidant and soybean lipoxygenase inhibitory activity.

## 2. Results and Discussion

**Scheme 1:**
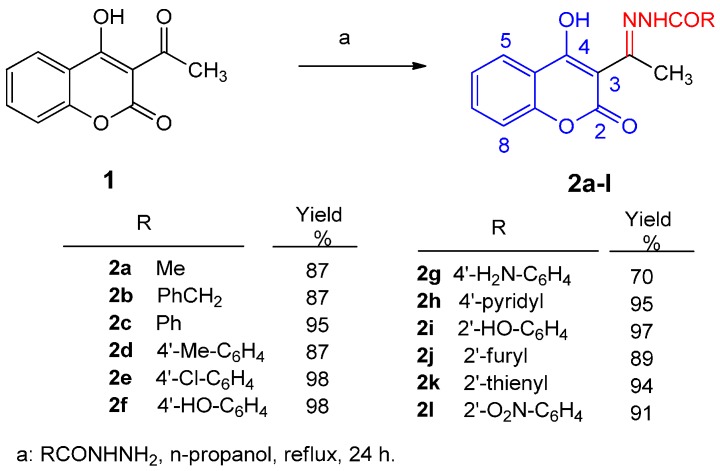
Synthesis of 3-acetyl-4-hydroxycoumarin *N*-acylhydrazones **2a**–**l**.

3-Acetyl-4-hydroxycoumarin *N*-acylhydrazones **2a**–**l** were prepared according to the literature [28] via treatment of 3-acetyl-4-hydroxycoumarins **1** with the appropriate hydrazide in *n*-propanol, as it is depicted in Scheme 1. The molar ratio of the reactants was 1:1. The reaction was performed under reflux for 24 h to yield hydrazones **2a**–**l** in excellent yields. Products **2b**, **2f**, **2g**, and **2h** are new compounds, whereas **2a**, **2c**–**2e**, and **2i**–**2l** have been recently synthesized and identified [28]. Compound **2a** has also been mentioned in the literature earlier [33] but its spectral data have been only recently reported [28]. New hydrazones **2b**, **2f**, **2g,** and **2h** were obtained in 70%–94%, whereas they have been alternatively afforded via reflux of ketone 1 with the appropriate hydrazide for 2 h in very good to excellent yields (69%–94%), comparatively lower to those under 24 h reflux (70%–98%). Hydrazones **2a**–**l** were purified via recrystallization from *n*-propanol. The mother ketone **1** has been prepared according to the literature by direct acetylation of 4-hydroxy-coumarin with acetyl chloride [34].

The structure of the new compounds **2b**, **2f**, **2g**, and **2h** was identified by their ^1^H- and ^13^C-NMR data as well as by their mass spectra and either their elemental analysis or high-resolution exact mass measurement. In ^1^H-NMR a characteristic singlet at about 2.65–2.77 ppm is assigned to the methyl protons attached at the 3-C=N carbon, whereas the proton at C-5 of the coumarin appears as a doublet of doublets at about 7.95 to 8.02 ppm in accordance with the literature data for other 4-hydroxycoumarin derivatives [29,35]. Furthermore, full assignments of the proton and carbon chemical shifts were based on coupling constants and on analogous coumarin derivatives assigned by detailed study of their 2D NMR data [35,36].

**Figure 1:**
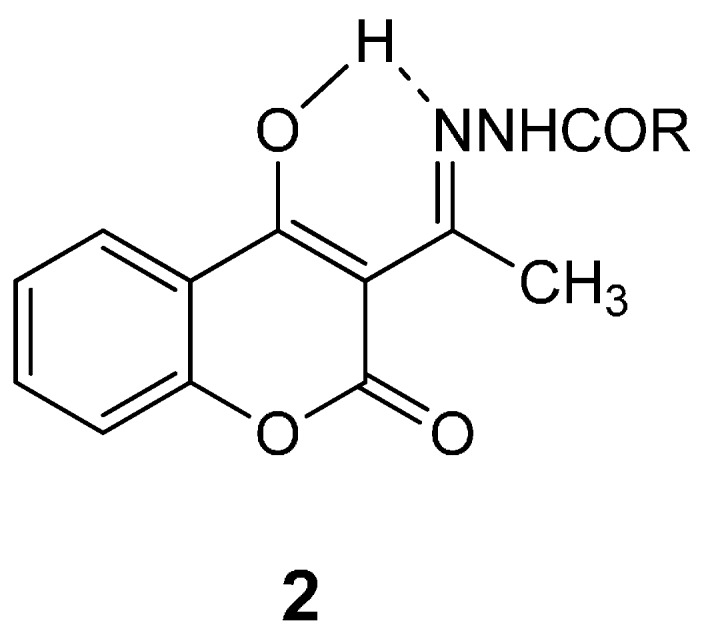
Hydrogen bond in 3-acetyl-4-hydroxycoumarin *N*-acylhydrazones.

Furthermore, hydrazones **2** show prominent peaks corresponding to the ion [M + 1] in their mass spectra. It should be noted that according to the literature data [35] compound **2** derivatives possibly exist as enols stabilized by hydrogen bond (as shown in Figure 1). Recently, the structure of 3-{*N*-[(2′-thienylcarbonyl)hydrazono]ethyl}-4-hydroxycoumarin **2k** has been confirmed by X-ray analysis [37].

### 2.1. Pharmacology

#### Antioxidant Activity

Taking the multifactorial character of oxidative stress into account, we decided to evaluate the *in vitro* antioxidant activity of the synthesized molecules using two different antioxidant assays. Therefore, the radical scavenging ability of the compounds was tested against the 2,2-diphenyl-1-picrylhydrazyl (DPPH) stable free radical and their ability to inhibit lipid peroxidation induced by the thermal free radical producer 2,2′-azobis(2-amidinopropane) dihydrochloride (AAPH) was evaluated.

**Figure 2:**
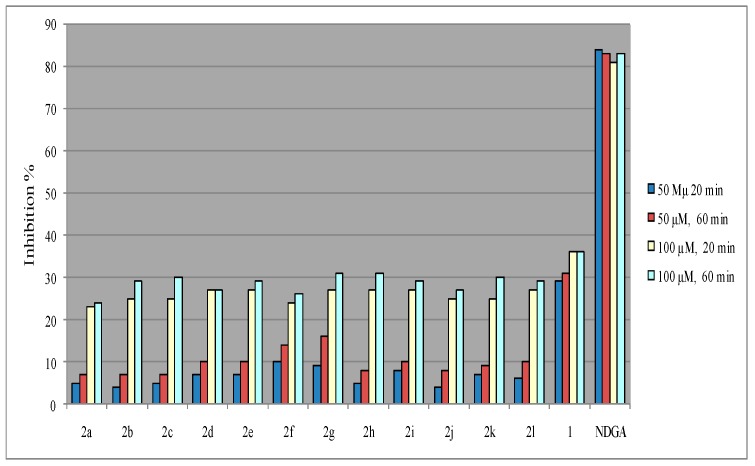
Effect of compounds **2** towards 2,2-diphenyl-2-picrylhydrazyl (DPPH).

**Scheme 2:**
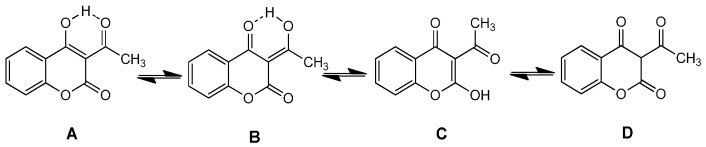
Tautomers (**A**–**D**) of 3-acetyl-4-hydroxycoumarin **1**.

It is well known that the interaction of the synthesized 3-acetyl-4-hydroxycoumarin *N*-acylhydrazones **2** with the stable free radical DPPH indicates their radical scavenging ability in an iron-free system [29]. In the present case, the interaction of the tested 3-acylhydrazono substituted 4-hydroxycoumarins **2** with DPPH was found to be concentration-dependent whereas, the time did not influence the reducing radical scavenging ability. Furthermore, all the tested compounds at 100 μΜ have presented similar radical reducing abilities ranging from 23%–27% to 24%–31% for 20 and 60 min respectively whereas, the interaction was found to be rather limited for the concentration of 50 μM (as shown in Figure 2 and in the collective Table 1). Considering the antioxidant activity of 3-acetyl-4-hydroxy coumarin (**1**) it seems to be higher than the hydrazone derivatives **2a**–**l** in both concentrations and in relation with the time and it is correlated with the presence of 3-acetyl and 4-hydroxy groups in the lactone ring and the possibility of tautomers (A–D) formation [38,39,40] as shown in Scheme 2. It has been reported that 3-acetyl-4-hydroxycoumarin mainly exists in endocyclic enol form (B) in polar solvents (methanol, ethanol) and it is well known that enols show antioxidant activity e.g., it has been reported that enolic and phenolic hydroxyl groups is beneficial for curcumin to protect erythrocytes against hemin-induced hemolysis and to protect DNA against AAPH-induced oxidation [41]. The lower results of hydrazones **2a**–**l**, are correlated with their stereochemistry which influence their interaction with DPPH.

**Table 1:** Inhibition % of DPPH at different concentrations and times, calculated lipophilicity Clog P [41] and % inhibition of LP and (LOX) (IC_50_) for compound **2**.

Compd.	RA%, 50 μM, 20 min	RA%, 50 μM, 60 min	RA%, 100 µM, 20 min	RA%, 100 μM, 60 min	Clog P	LP ^a^ 60 s, 100 μM	LOX ^b^ IC_50_ (μM)
**2a**	5 ± 0.2	7 ± 0.3	23 ± 3.0	24 ± 2.0	1.85	100 ± 9.8	62.5 ± 2.3
**2b**	4 ± 0.2	7 ± 0.1	25 ± 2.0	29 ± 1.1	3.62	100 ± 5.5	40 ± 0.5
**2c**	5 ± 0.1	7 ± 0.4	25 ± 1.2	30 ± 2.8	3.29	98 ± 5.4	58 ± 2.7
**2d**	7 ± 0.3	10 ± 0.2	27 ± 2.2	27 ± 1.4	3.79	100 ± 3.2	No ^c^
**2e**	7 ± 0.2	10 ± 0.5	27 ± 0.9	29 ± 0.8	4.20	94 ± 4.8	55 ± 2.1
**2f**	10 ± 0.5	14 ± 1.2	24 ± 2.2	26 ± 1.2	2.96	98 ± 2.9	70 ± 4.3
**2g**	9 ± 0.3	16 ± 0.6	27 ± 0.8	31 ± 1.4	2.38	95 ± 7.2	46.5 ± 2.3
**2h**	5 ± 0.1	8 ± 0.2	27 ± 1.5	31 ± 1.6	2.53	99 ± 3.7	No ^c^
**2i**	8 ± 0.5	10 ± 0.1	27 ± 0.2	29 ± 1.7	2.96	100 ± 8.2	49.5 ± 1.2
**2j**	4 ± 0.2	8 ± 0.3	25 ± 1.8	27 ± 0.8	2.46	95 ± 4.1	90 ± 5.1
**2k**	7 ± 0.1	9 ± 0.2	25 ± 2.1	30 ± 2.2	3.13	98 ± 3.9	43.5 ± 3.2
**2l**	6 ± 0.3	10 ± 0.2	27 ± 2.2	29 ± 1.0	3.46	95 ± 6.2	35 ± 0.2
**1**	29 ± 0.5	31± 0.3	36± 1.3	36 ± 0.8	1.91	8 ± 0.2	44 (± 0.3) ^d^
**NDGA**	84 ± 2.0	83 ± 3.3	81 ± 5.2	83 ± 4.7			5.5 ± 0.1
**TROLOX**						63 ± 0.2	

^a^ % inhibition of LP induced by AAPH; ^b^ *in vitro* inhibition of soybean lipoxygenase (LOX); ^c^ no action under the reported experimental conditions; ^d^ the presented biological response is given as % inhibition. The IC_50_ value was not be able to be determined.

AAPH induced linoleic acid oxidation is based on the inhibition of lipid oxidation and provides a measure of how efficiently antioxidants protect against lipid oxidation *in vitro*. Oxidation of exogenous linoleic acid by a thermal free radical producer (AAPH) is followed by UV spectrophotometry in a highly-diluted sample [36,42].

**Figure 3:**
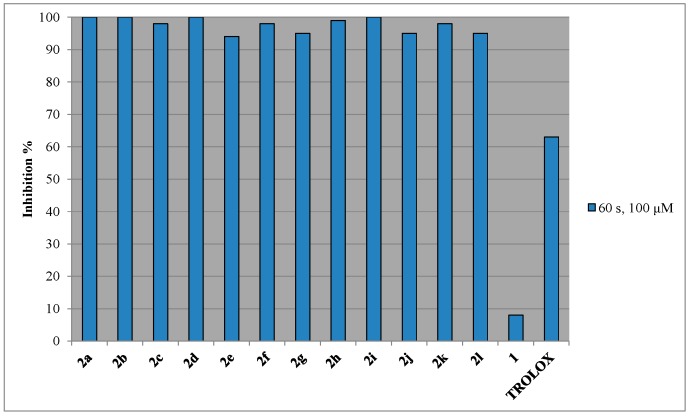
Effect of compound **2** towards AAPH lipid peroxidation.

In general, all the studied compounds effectively inhibit AAPH induced lipid peroxidation, showing higher activity than the reference compound trolox (63%, Figure 3 and Table 1). 3-Acetyl-4-hydroxy coumarin (1) presents non-significant anti-lipid peroxidation activity. However, all the derivatives exhibit very potent inhibition of lipid peroxidation and almost the same as a result of their combined structural characteristics. Lipophilicity does not seem to play any significant role. For example, methyl derivative **2a**, is a good inhibitor of lipid peroxidation (100%), while it presents the lowest *Clog P* value among all the analogues (Table 1). The tested derivatives possess a favorable electronic distribution for reacting quickly with intermediate lipid peroxy radicals and sufficient lipid solubility to partition effectively in lipid bilayers. Our preliminary results suggest that they are indeed capable to inactivate rapidly alkylperoxy radicals.

### 2.2. In Vitro Inhibition of Soybean Lipoxygenase (LOX)

**Figure 4:**
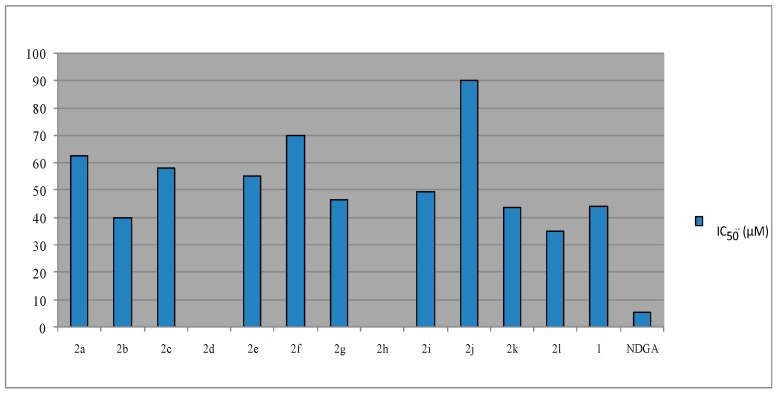
Effect of compounds **2** towards soybean lipoxygenase (LOX).

Coumarins as well as flavonoids are among the most potent 5-lipoxygenase inhibitors. The synthesized coumarins were evaluated for inhibition of soybean lipoxygenase by the UV-absorbance-based enzyme assay [29] and the results are presented in Figure 4 as well as in Table 1. The majority of LOX inhibitors are antioxidants or free radical scavengers [43], since lipoxygenation occurs via a carbon-centered radical. Some studies suggest a relationship between LOX inhibition and the ability of the inhibitors to reduce the Fe^3+^ at the active site to the catalytically inactive Fe^2+^. Several LOX inhibitors are excellent ligands for Fe^3+^ [44,45]. It has been demonstrated that their mechanism of action is presumably related to their coordination with a catalytically crucial Fe^3+^. 3-Acetyl-4-hydroxy coumarin (1) showed low inhibitory activity at 100 µM and, thus, we did not proceed to determine its IC_50_ value. In Table 1, its response is given, as a % inhibition value at 100 µM. Among the tested compounds, the 2′-NO_2_-substituted phenyl (**2l**) was found to exhibit superior LOX inhibitory activity, followed by the benzyl substituted hydrazine (**2b**), the 2′-thienyl-substituted derivative (**2k**), the 4-NH_2_-phenyl substituted hydrazine (**2g**) and the 2′-OH-substituted phenyl (**2i**) (Figure 4, Table 1). No sign for the role of overall lipophilicity is obvious. However, the three most potent derivatives **2l**, **2b**, and **2k** present a mean value of Clog P = 3.4. The 2′-thienyl-substituted derivative (**2k**) is more potent than the corresponding 2’-furyl derivative (**2j**), whereas the 4’-pyridyl-analogue **2h** and the 4′-CH_3_-substituted phenyl hydrazone (**2d**) do not seem to present any activity under the reported experimental conditions. The position of substitution is significant since the 2-substituted derivative, e.g., the 2′-OH-substituted phenyl (**2i**) is more potent than the corresponding 2f which is a 4′-OH-substituted phenyl hydrazone. Small differences are observed when R is a phenyl or a small alkyl group. Each *in vitro* experiment was performed at least in triplicate and the standard deviation of absorbance was less than 10% of the mean.

## 3. Experimental Section

### 3.1. General

3-Acetyl-4-hydroxycoumarin **1** was synthesized according to the literature [28,34]. Melting points are uncorrected and were determined on a Fisher-Johns melting point apparatus. 1D-NMR spectra were recorded at room temperature on a Bruker Avance 400 spectrometer (Bruker, Billerica, MA, USA) at 400.15 MHz for ^1^H-NMR and 100.62 MHz for ^13^C-NMR in DMSO-*d*_6_. The chemical shifts are expressed in δ values (ppm) relative to TMS as internal standard for ^1^H and relative to TMS (0.00 ppm) or to DMSO-*d*_6_ (39.50 ppm) for ^13^C-NMR spectra. Coupling constants *^n^J* are reported in Hz. Second order ^1^H spectra, where it was possible, were analyzed by simulation [46]. Either elemental analysis or HRMS has been provided for all new products of **2**. All the chemicals used for biological assays were of analytical grade and commercially available by Merck, 2,2-diphenyl-1-picrylhydrazyl (DPPH), nordihydroguairetic acid (NDGA), trolox, and AAPH were purchased from the Aldrich Chemical Co. (Milwaukee, WI, USA). Soybean Lipoxygenase and linoleic acid sodium salt were obtained from Sigma Chemical, Co. (St. Louis, MO, USA).

### 3.2. Chemistry

#### Synthesis of 3-[1-(Acyl-hydrazono)ethyl]-4-hydroxycoumarins (**2a**–**l**)

To a solution of 3-acetyl-4-hydroxy-coumarin **1** (1 mmol) in *n*-propanol (15–20 mL) was added the appropriate hydrazide (1 mmol). The mixture was refluxed for 24 h and cooled at room temperature. The precipitate was collected by filtration and dried to give the 3-[1-(acyl-hydrazono)ethyl]-4-hydroxycoumarin (**2a**–**l**) as solid and was then recrystallized from *n*-propanol in very good yields. The following compounds have been prepared according to this procedure:

*3-[N-(Acetylhydrazono)ethyl]-4-hydroxycoumarin* (**2a**). Yield: 87% under reflux for 24 h and 81% under reflux for 2 h; light yellow solid; mp 248–249 °C (mp 250–251 °C (from MeOCH_2_CH_2_OH/H_2_O) [30]); ^1^H-NMR (DMSO-*d*_6_, 400 MHz) δ 2.07 (s, 3H, 3-COCH_3_), 2.66 (s, 3H, 3-CCH_3_), 7.29 (dd, *J* = 8.3, 1.0 Hz, 1H, 8-H), 7.32 (ddd, *J* = 7.9, 7.3, 1.0 Hz, 1H, 6-H), 7.66 (ddd, *J* = 8.3, 7.3, 1.8 Hz, 1H, 7-H), 7.97 (dd, *J* = 7.9 Hz, 1.8 Hz, 1H, 5-H), 11.42 (s, 1H, NNH), 15.90 (br s, 1H, 4-OH); ^13^C-NMR (DMSO-*d*_6_, 100 MHz) δ 17.3 (3-C*C*H_3_), 20.4 (3-CO*C*H_3_), 94.9 (C-3), 116.3 (C-8), 119.5 (C-4a), 123.8 (C-5), 125.5 (C-6), 134.2 (C-7), 153.0 (C-8a), 161.8 (C-2), 167.0 (3-C=N), 169.2 (NHCO), 178.7 (C-4); HRMS (ESI^+^) calcd for C_13_H_12_N_2_O_4_ *m*/*z*: 261.08698 (M + H^+^); found 261.08688 (M + H^+^).

*3-[N-(Phenylacetylhydrazono)ethyl]-4-hydroxycoumarin* (**2b**). Yield: 87% under reflux for 24 h and 83% under reflux for 2 h; yellow solid; mp 216–217 °C; ^1^H-NMR (DMSO-*d*_6_, 400 MHz) δ 2.65 (s, 3H, 3-CCH_3_), 3.70 (s, 2H, C*H_2_*Ph), 7.28 (dd, *J* = 8.3, 1.0 Hz, 1H, 8-H), 7.28–7.36 (m, 6H, 6-H, C_6_H_5_), 7.64 (ddd, *J* = 8.3, 7.3, 1.0 Hz, 1H, 7-H), 7.95 (dd, *J* = 7.8, 1.5 Hz, 1H, 5-H), 11.7 (s, 1H, NNH), 15.9 (br, 1H, 4-OH); ^13^C-NMR (DMSO-*d*_6_, 100 MHz) δ 17.8 (3-C*C*H_3_), 40.4 (CH_2_, masked under the septet of the solvent), 95.5 (C-3), 116.8 (C-8), 119.9 (C-4a), 124.3 (C-5), 126.0 (C-6), 127.3 (C-4′), 128.9 (C-3′,5′), 129.7 (C-2′,6′), 134.7 (C-7), 135.2 (C-1′), 153.5 (C-8a), 161.8 (C-2), 168.4 (3-C=N), 170.3 (NHCO), 179.2 (C-4); HRMS (ESI^+^) calcd for C_19_H_16_N_2_O_4_ *m*/*z*: 359.10023 (M + Na^+^), 695.21124 (2M + Na^+^); found 359.10031 (M + Na^+^), 695.21160 (2M + Na^+^).

*3-[N-(Benzoylhydrazono)ethyl]-4-hydroxycoumarin* (**2c**). Yield: 95%; white solid; mp 225–226 °C [28] 

*3-{N-[(4′-Methylbenzoyl)hydrazono]ethyl}-4-hydroxycoumarin* (**2d**). Yield: 87%; white solid; mp 251–252 °C [28].

*3-{N-[(4′-Chlorobenzoyl)hydrazono]ethyl}-4-hydroxycoumarin* (**2e**). Yield: 98%; white solid; mp 248–248.5 °C [28].

*3-{N-[(4*′*-Hydroxybenzoyl)hydrazono]ethyl}-4-hydroxycoumarin* (**2f**). Yield: 98% under reflux for 24 h and 94% under reflux for 2 h; white solid; mp 287–288 °C; ^1^H-NMR (DMSO-*d_6_*, 400 MHz) δ 2.75 (s, 3H, 3-CCH_3_), 6.91 (d, *J* = 8.7 Hz, 2H, 3′,5′-H), 7.30 (dd, *J* = 8.3, 1.0 Hz, 1H, 8-H), 7.33 (ddd, *J* = 7.8, 7.2, 1.0 Hz, 1H, 6-H), 7.67 (ddd, *J* = 8.3, 7.0, 1.7 Hz, 1H, 7-H), 7.84 (d, *J* = 8.7 Hz , 2H, 2′,6′-H), 8.00 (dd, *J* = 7.9, 1.7 Hz, 1H, 5-H), 10.34 (br s, 1H, 4′-OH), 11.55 (s, 1H, NNH), 15.72 (br, 1H, 4-OH); ^13^C-NMR (DMSO-*d*_6_, 100 MHz) δ 18.1 (3-C*C*H_3_), 95.7 (C-3), 115.8 (C-3′,5′), 116.8 (C-8), 120.2 (C-4a), 122.0 (C-1′), 124.3 (C-5), 126.2 (C-6), 130.6 (C-2′,6′), 134.8 (C-7), 153.6 (C-8a), 161.98 (C-4′), * 162.04 (C-2), * 165.0 (3-C=N), 172.2 (NHCO), 179.7 (C-4); MS (ESI): *m*/*z* 338 (M^+^). Anal. calcd for C_18_H_14_N_2_O_5_: C, 63.90; H, 4.17; N, 8.28; found C, 63.70; H, 3.98; N, 8.44. (*: The assignments may be interchanged).

*3-{N-[(4*′*-Aminobenzoyl)hydrazono]ethyl}-4-hydroxycoumarin* (**2g**). Yield: 70% under reflux for 24 h and 69% under reflux for 2 h; light yellow solid; mp 256–257 °C; ^1^H-NMR (DMSO-*d*_6_, 400 MHz) δ 2.74 (s, 3H, 3-CCH_3_), 6.01 (br, 2H, NH_2_), 6.63 (d, *J* = 8.6 Hz, 2H, 3′,5′-H), 7.29 (d, *J* = 8.4 Hz, 1H, 8-H), 7.33 (dd, *J* = 7.8, 7.2 Hz, 1H, 6-H), 7.66 (ddd, *J* = 8.4, 7.2, 1.5 Hz, 1H, 7-H), 7.69 (d, *J* = 8.6 Hz, 2H, 2′,6′-H), 8.00 (dd, *J* = 7.8, 1.5 Hz, 1H, 5-H), 11.30 (s, 1H, NNH), 15.74 (br, 1H, 4-OH); ^13^C-NMR (DMSO-*d*_6_, 100 MHz) δ 18.2 (3-C*C*H_3_), 95.5 (C-3), 113.2 (C-3′,5′), 116.8 (C-8), 117.2 (C-1′), 120.3 (C-4a), 124.3 (C-5), 126.1 (C-6), 130.3 (C-2′,6′), 134.6 (C-7), 153.6 (C-8a), * 153.8 (C-4′), * 162.0 (C-2), 165.2 (3-C=N), 171.4 (NHCO), 179.6 (C-4); MS (ESI) *m*/*z* 337 (M^+^). Anal. Calcd for C_18_H_15_N_3_O_4_ C, 64.09; H, 4.48; N, 12.46. Found C, 63.73; H, 4.25; N, 12.46. (*: The assignments may be interchanged).

*3-[N-(Isonicotinoylhydrazono)ethyl]-4-hydroxycoumarin* (**2h**). Yield: 95% under reflux for 24 h and 91% under reflux for 2 h; orange solid; mp 274 °C; ^1^H-NMR (DMSO-*d*_6_, 400 MHz) δ 2.78 (s, 3H, 3-CCH_3_), 7.31 (dd, *J* = 8.3, 1.0 Hz, 1H, 8-H), 7.34 (ddd, *J* = 7.8, 7.4, 1.0 Hz, 1H, 6-H), 7.67 (ddd, *J* = 8.3, 7.4, 1.6 Hz, 1H, 7-H), 7.95 (br d, *J* = 4.8 Hz, 2H, 2′,6′-H), 8.01 (dd, *J* = 7.9, 1.6 Hz, 1H, 5-H), 8.84 (br s, 2H, 3′,5′-H), 11.9 (br, 1H, NNH), 15.75 (br s, 1H, 4-OH); ^13^C-NMR (DMSO-*d*_6_, 100 MHz) δ 17.6 (3-C*C*H_3_), 95.3 (C-3), 116.3 (C-8), 119.9 (C-4a), 122.0 (C-2′,6′), 123.8 (C-5), 125.6 (C-6), 134.2 (C-7), 140.6 (br, C-1′), 149.3 (br, C-3′,5′), 153.1 (C-8a), 161.50 (C-2), 163.2 (3-C=N), 171.0 (br, NHCO), 178.9 (C-4); HRMS (ESI^+^) Anal. Calcd for C_17_H_13_N_3_O_4_ *m*/*z*: 324.09788 (M + H^+^); Found 324.09784 (M+H^+^).

*3-{N-[(2′-Hydroxybenzoyl)hydrazono]ethyl}-4-hydroxycoumarin* (**2i**). Yield: 97%; white solid; mp 271–272 °C [28].

*3-{N-[(2*′*-Furoyl)hydrazono]ethyl}-4-hydroxycoumarin* (**2j**). Yield: 89%; yellow solid; mp 254.5–255.0 °C [28].

*3-{N-[(2*′*-Thienylcarbonyl)hydrazono]ethyl}-4-hydroxycoumarin* (**2k**). Yield: 94%; light yellow solid; mp 228–228.5 °C [28].

*3-{N-[(2′-Nitrobenzoyl)hydrazono]ethyl}-4-hydroxycoumarin* (**2l**). Yield: 91%; yellow solid; mp 219 °C [28].

### 3.3. Pharmacology

#### 3.3.1. Determination of the Reducing Activity of the DPPH (RA%)

To an ethanolic solution of DPPH (0.05 mM) in absolute ethanol the new coumarin derivatives dissolved in DMSO were added (final concentration 50 and 100 µM). The mixture was shaken vigorously and allowed to stand for 20 min or 60 min; absorbance at 517 nm was determined spectrophotometrically against the blank and the percentage of reducing activity (RA) was calculated by the formula: RA% = [(A_0_ − A_1_)/A_0_] × 100 where A_0_ is the absorbance of blank and A_1_ is the absorbance of the reaction mixture. All tests were undertaken on three replicates and the results presented in Table 1 were averaged.

#### 3.3.2. Inhibition of Linoleic Acid Lipid Peroxidation

Production of conjugated diene hydroperoxide by oxidation of linoleic acid in an aqueous dispersion is monitored at 234 nm. 2,2′-Azobis(2-amidinopropane) dihydrochloride (AAPH) is used as a free radical initiator. Ten microliters of the 16 mM linoleic acid sodium salt solution was added to the UV cuvette containing 930 µL of 0.05 mM phosphate buffer, pH 7.4 prethermostated at 37 °C. The oxidation reaction was initiated at 37 °C under air by the addition of 50 μL of 40 mM AAPH solution. Oxidation was carried out in the presence of aliquots (10 μL) of the tested coumarins. In the assay without antioxidant, lipid peroxidation was measured in the presence of the same level of DMSO. The rate of oxidation at 37 °C was monitored by recording the increase in absorption at 234 nm caused by conjugated diene hydroperoxides.

#### 3.3.3. Soybean Lipoxygenase Inhibition Study *In Vitro*

The tested compounds dissolved in DMSO were incubated at room temperature with sodium linoleate (100 µL) and 200 µL of enzyme solution (1/9 × 10^−4^ *w*/*v* in saline) in Tris buffer pH 9. The conversion of sodium linoleate to 13-hydroperoxylinoleic acid at 234 nm was recorded and compared with the appropriate standard inhibitor.

#### 3.3.4. Physicochemical Studies

Since lipophilicity is a significant physicochemical property determining distribution, bioavailability, metabolic activity, and elimination, the theoretically calculated [47] *Clog P* values in *n*-octanol-buffer are included in Table 1. For their determination the C-QSAR program of Biobyte Corp. was used.

## 4. Conclusions

In this study, a series of 3-acylhydrazono substituted 4-hydroxycoumarins have been synthesized and characterized. The antioxidant activity of the synthesized compounds has been studied *in vitro* using two different assays. Moreover, in an attempt to identify the potential of the compounds as anti-inflammatory agents, their ability to inhibit *in vitro* soybean lipoxygenase was evaluated. Although the free 4-hydroxy coumarin was not found to present any antioxidant activity [48] its combination with a 3-imino group [49] recently led to antioxidant properties. These results go in parallel to our findings, where the combination of 4-hydroxy coumarin with the 3-acyl-hydrazone group leads to potent inhibitors of lipid peroxidation.

Our study indicates that high LOX inhibitory activity is not accompanied by high DPPH radical scavenging activity. This is in accordance with the finding of Curini *et al.* [50], who have studied the antioxidant and LOX inhibitory activity of five natural prenyloxy-carboxylic acids and showed that the most efficient LOX inhibitor (boropinic acid) is not the most active DPPH radical scavenger. However, a better correlation exists between LOX inhibitory activity and lipid peroxidation inhibition.

It is of interest that compound **2l**, the 2-nitro-substituted-3-acylhydrazono-4-hydroxy-coumarin, is the most potent as it possesses an array of potentially beneficial characteristics: it inhibits lipid peroxidation with satisfactory potency and it inhibits LOX (IC_50_ = 35 μM). It would, thus, be of special interest to characterize this molecule in terms of its anti-inflammatory profile.
